# Pleural Kaposi Sarcoma in Two HIV-Positive Patients

**DOI:** 10.7759/cureus.64938

**Published:** 2024-07-19

**Authors:** Douaa Abou Hamdan, Ghadir Hayek, Pierre Abi Hanna, Michelle Saliba, Issam Chehade, Rozan Youssef, Farah Assi

**Affiliations:** 1 Infectious Diseases, Lebanese University Faculty of Medical Sciences, Beirut, LBN; 2 Infectious Disease, Lebanese University Faculty of Medical Sciences, Beirut, LBN; 3 Infectious Diseases, Rafic Hariri University Hospital, Beirut, LBN; 4 Hematology and Medical Oncology, Rafic Hariri University Hospital, Beirut, LBN; 5 Internal Medicine, Lebanese University Faculty of Medical Sciences, Beirut, LBN; 6 Infectious Diseases, Saint George Hospital University Medical Center, Beirut, LBN

**Keywords:** aids-defining illness, hiv, liposomal doxorubicin, haart, pleural kaposi sarcoma

## Abstract

Kaposi sarcoma is a neoplasm caused by human herpes virus 8 (HHV-8) that commonly presents as subcutaneous lesions but can also involve visceral organs such as the gastrointestinal and pulmonary systems. Diagnosis is achieved through histopathological analysis of cutaneous lesions or lymph nodes. In this study, we report two patients, recently diagnosed with HIV, who were later found to have cutaneous and visceral (pleural) Kaposi sarcoma. In both cases, the patients presented with dyspnea accompanied by cutaneous lesions and bilateral pleural effusion. Unfortunately, the first patient did not survive long enough for treatment initiation. The second patient, however, demonstrated a favorable response to a treatment regimen comprising highly active antiretroviral therapy (HAART) and liposomal doxorubicin.

## Introduction

Kaposi sarcoma (KS) is a low-grade angioproliferative neoplasm characterized by the development of multiple nodules affecting the skin, mucous membranes, and internal organs [1]. Human herpes virus-8 (HHV-8), also called KS-associated herpesvirus, has been identified as the causative agent of KS, and it has been demonstrated to exist in every clinical variant of KS [2]. KS exhibits significant morbidity and mortality and ranks globally as the most common HIV-related malignancy [3]. While cutaneous and mucous membrane manifestations are prevalent, cases of visceral localization have been documented [4]. Visceral KS most commonly involves gastrointestinal (GI) sites and lymph nodes [5]. Occurrence of visceral KS in the respiratory tract is an uncommon initial presentation and, in over 90% of the cases, extensive mucocutaneous disease is present as well [6]. In this report, we present two cases of pleural involvement of KS in the setting of recently diagnosed advanced HIV/AIDS. Both patients were of Levantine origin (Lebanese).

## Case presentation

Case 1

A 54-year-old male presented to the emergency department with complaints of increasing cough, dyspnea, orthopnea, and generalized weakness. His complaints started two months ago when he experienced significant weight loss and lower limb edema. Outpatient workup revealed left leg superficial thrombophlebitis and multiple enlarged intra-abdominal lymph nodes with bilateral pleural effusions. A submandibular lymph node biopsy revealed Piringer-Kuchinka lymphadenitis, suggestive of toxoplasmosis. An underlying immunocompromise was confirmed with a positive HIV antibody/antigen (Ab/Ag) test (CD4 count 231 cells/mm^3^; viral load 25,450 RNA copies/ml). The patient presented to our institution before the initiation of HAART.

**Figure 1:**
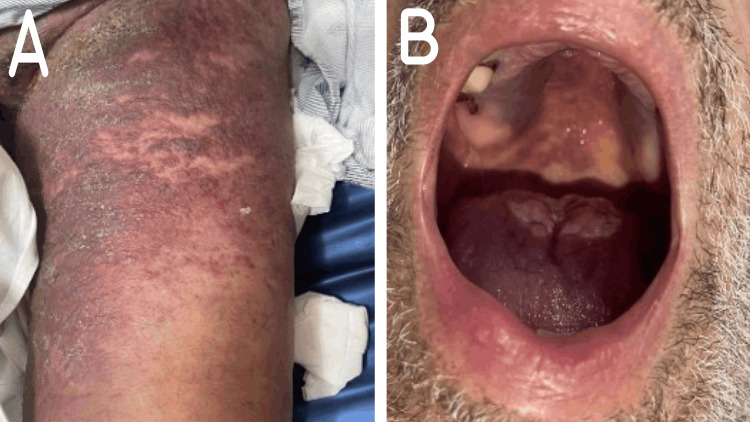
Mucocutaneous lesions of Kaposi sarcoma in Case 1. (A) skin lesions on the lower extremity and pelvic area; (B) oral mucosa lesions on the hard palate.

Upon admission, he appeared ill, and dyspneic, though hemodynamically stable on room air. The physical exam revealed bilateral decreased air entry, lower limb edema, and cutaneous lesions extending from the thighs to the pelvic area. The lesions were non-tender, palpable, and reddish purplish, with areas of overlying crusting (Figure 1A). Inspection of the oral cavity displayed a similar purplish lesion on the hard palate, with a whitish discoloration on the tongue (Figure 1B).

**Figure 2:**
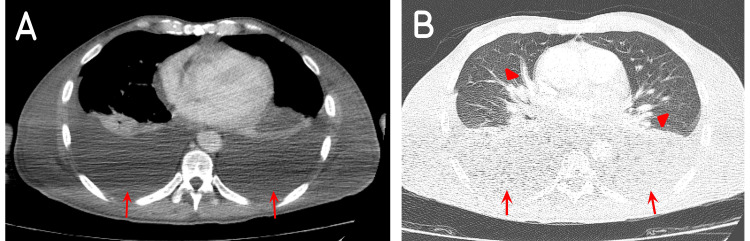
CECT of the chest in Case 1 showing (A, B) a moderate amount of bilateral pleural effusions (red arrows) and (B) congested lung parenchyma (red arrowheads). A, mediastinal view; B, lung view CECT: contrast-enhanced computed tomography

Initial blood tests revealed a white blood cell count (WBC) of 7,000/mL (4,000-11,000/mL), a hemoglobin level of 12 g/dl (14-16 g/dl), a creatinine level of 1.2 mg/dl (0.2-1.2 mg/dl), and a C-reactive protein (CRP) level of 33 mg/L (< 5 mg/L).The patient was admitted to the regular floor, and started on ceftriaxone 2 g intravenously (IV) once daily, azithromycin 500 mg orally once daily, and fluconazole 200 mg orally once daily. A total body contrast-enhanced computed tomography scan (CECT) was performed aiming to rule out brain lesions and pulmonary embolism. None were discovered. Multiple bone, liver, and pancreatic head lesions were seen on abdominal view, with retroperitoneal lymphadenopathy. At the same time, a moderate amount of bilateral pleural effusion, and congested lung parenchyma were seen on chest imaging (Figure 2). Visceral KS was suspected.

On the sixth day of admission, the patient developed febrile septic shock and was transferred to the intensive care unit (ICU) on vasopressor support. The cutaneous lesions on the lower extremities were oozing purulent materials. His antibiotics were switched to meropenem and vancomycin. Blood cultures yielded no bacterial growth, but the pus culture revealed multi-drug resistant (MDR) *Acinetobacter baumannii* and *Klebsiella pneumoniae*. Pleural fluid was obtained for analysis, culture, and further studies. Results are displayed in Table 1.

**Table 1:** Pleural fluid analysis results in Case 1. RBC, red blood cells; WBC, white blood cells; PMNs, polymorphonuclear cells; PCR, polymerase chain reaction; HHV, human herpes virus

Parameter	Finding	Normal ranges
RBC	29000	0-5 cells/µ
WBC	800	0-250 cells/µ
PMNs	45%	-
Lymphocytes	47%	-
Monocytes	8%	-
Mycoplasma bacteria PCR	Undetectable	Undetectable
HHV-6 PCR	Undetectable	Undetectable
HHV-7 PCR	Undetectable	Undetectable
HHV-8 PCR	Positive	Undetectable

Given the results, the diagnosis of pleural and cutaneous KS was established. A prompt decision to start treatment with liposomal doxorubicin in combination with HAART was undertaken; however, the patient's condition continued rapidly deteriorating, leading to the development of severe acute respiratory distress syndrome (ARDS). Ultimately, the patient passed away.

Case 2

A 31-year-old male presented to our institution complaining of fever, left lower limb edema, progressive dyspnea, and pleuritic chest pain of two weeks’ duration. Two weeks prior to presentation the patient underwent a biopsy of an inflamed left cervical lymph node that had been enlarged for the duration of two years. The biopsy result was positive for HHV-8 and consistent with KS. Three days prior to presentation, polymerase chain reaction (PCR)-HIV test was performed and found to be positive (HIV viral load 289,000 RNA copies/ml, CD4 count of 663 cells/mm^3^).

**Figure 3:**
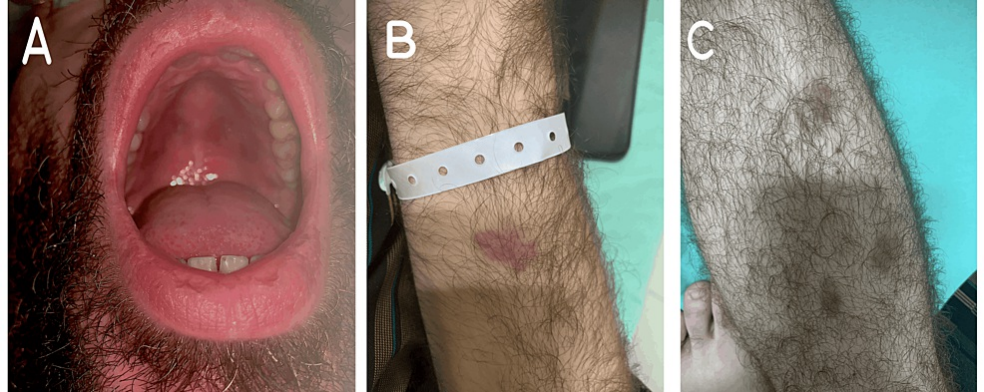
Mucocutaneous lesions of Kaposi sarcoma in Case 2. (A) Oral mucosal lesions on the hard palate; (B) Cutaneous lesions on the upper extremity; (C) Cutaneous lesions on the lower extremity.

Upon presentation, he appeared dyspneic, with decreased bilateral air entry on auscultation. There were multiple bilaterally palpable lymph nodes on the inguinal areas, with an edematous and erythematous left leg. Multiple non-tender cutaneous reddish-purplish lesions were seen in the oral cavity (Figure 3A), as well as on the upper and lower extremities (Figure 3B, 3C).

**Figure 4:**
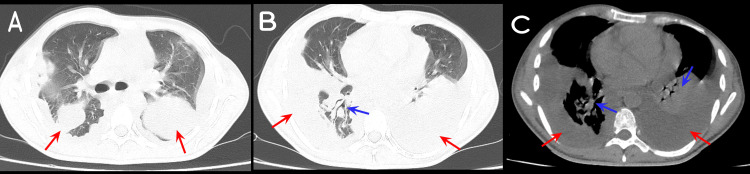
Non-enhanced CT scan of the chest in Case 2 showing (A, B, C) a moderate amount of bilateral loculated pleural effusion (red arrows) with (B, C) bilateral lung consolidation (blue arrows). A, B, lung view; C, mediastinal view

Initial blood tests revealed leukocytosis, with WBC count of 13,000/mL (4,000-11,000/mL) and left shift (83% neutrophils), hemoglobin level of 12 g/dl (14-16 g/dl), creatinine level of 1.4 mg/dl (0.2-1.2 mg/dl), and CRP level > 380 mg/L (< 5 mg/L). A venous Doppler ultrasound of bilateral lower extremities showed no thrombosis. A non-enhanced CT scan of the chest, abdomen, and pelvis showed a moderate amount of loculated bilateral pleural effusion with bilateral consolidation (Figure 4), hepatomegaly (18 cm), multiple enlarged scattered intra-abdominal and aortic lymph nodes, and moderate abdominal ascites.

The patient was admitted to the regular floor, and started on ceftriaxone 2 g IV daily, empirically, targeting lower respiratory tract infection, and left leg cellulitis. The differential diagnosis included active *Pneumocystis pneumonia* infection and pulmonary involvement of KS. Oral trimethoprim-sulfamethoxazole at the therapeutic dose of 20 mg/kg/day was initiated. Pleural fluid was obtained for analysis, culture, and viral PCR. The aspirated fluid was turbid, and the analysis exhibited an exudative quality. The results are displayed in Table 2.

**Table 2:** Pleural fluid analysis results in Case 2. RBC, red blood cells; WBC, white blood cells; LDH, lactate dehydrogenase; HHV, human herpes virus; PCR, polymerase chain reaction; RT-PCR, real-time polymerase chain reaction

Parameter	Finding	Normal ranges
RBC	940	0-5 cells/µ
WBC	200	0-250 cells/µ
Glucose	20	30-60 mg/dl
LDH	> 2,100	0-200 IU/L
Proteins	64	0-4 g/dl
HHV-8 PCR	Detected	Undetectable
HHV-6 PCR	Undetectable	Undetectable
HHV-7 PCR	Undetectable	Undetectable
*Mycobacterium tuberculosis* PCR	Undetectable	Undetectable
*Pneumocystis pneumonia* RT-PCR	Undetectable	Undetectable

The patient’s WBC count further increased, and his antibiotics were escalated to meropenem (2 g IV every eight hours). He remained hemodynamically stable on room air. The diagnosis of pleural KS was established. On the 10th day of admission, HAART was initiated. The treatment consisted of elvitegravir 150 mg/cobicistat 150 mg/emtricitabine 200 mg/tenofovir alafenamide 10 mg, followed by liposomal doxorubicin (35 mg IV in 250 ml of dextrose water via a one-hour infusion) on day 12 of admission.

The patient was monitored for nine days post chemotherapy, and he displayed a favorable course. He was discharged on scheduled appointments to continue treatment. Upon discharge, his WBC count, creatinine, and CRP levels came within normal ranges. Later, the patient completed six cycles of liposomal doxorubicin with commendable tolerance and achieved excellent therapeutic outcomes. A follow-up CECT scan demonstrated regression of all abdominal and aortic lymphadenopathies and resolution of his ascites. After six months, the patient was lost to follow-up in the clinic. After a year, it was discovered that he had stopped all his medications, shortly after which he passed away due to complications.

## Discussion

KS, a lymphangioproliferative tumor related to HHV-8 infection, occurs typically in immunocompromised states [1]. Although KS can develop at any stage of HIV infection, it occurs more commonly in the setting of advanced immune suppression [7]. KS exhibits four epidemiological variants: classic, African (endemic), immunosuppression-related (iatrogenic), and epidemic or AIDS-related KS [5,7]. The classic form rarely portrays systemic progression and is usually characterized by solitary cutaneous lesions on the distal extremities [8]. The endemic type is HIV-independent and comprises two entities: cutaneous and lymphadenopathic [7]. The immunosuppression-related KS affects individuals following organ transplantations or in any state of immunosuppression [9]. This variant appears to undertake a more aggressive course and occasionally occurs without cutaneous involvement [9]. The final variant is associated with AIDS and is termed the epidemic form. Aggressive and frequently fatal, AIDS-related KS may present with localized or disseminated skin lesions and may involve GI and visceral sites [7,9].

In AIDS-related KS, the CD4 count carries an inverse relationship with the development of KS, with a higher rate ratio of developing KS in patients with CD4 counts < 200 cells/µL [6]. However, in a study involving 466 patients with AIDS-related KS, fewer than half had KS diagnosis at a CD4 count < 200 cells/µL, 15% at a CD4 count ≥ 500 cells/µL, and 29% had suppressed HIV viral load (500 copies/ml) [10]. This change can be attributed to the early institution of HAART upon HIV diagnosis and reflects an improvement of healthcare systems [10].

Any organ can be subjected to the development of KS [4]. The most common extra-cutaneous location of KS in HIV/AIDS patients is GI KS, with 75% of the affected patients typically lacking symptoms at initial presentation [1,11]. When symptomatic, they present with abdominal pain, GI bleeding, diarrhea, and obstruction [11]. Respiratory symptoms include hypoxemia, nonproductive cough, hemoptysis, weight loss, and fever, indiscernible from other AIDS-related opportunistic lung diseases, thus posing a diagnostic challenge [12]. Pleural effusions are a frequently encountered finding in imaging of KS, especially the pulmonary variant, which most characteristically presents with peri-bronchial and perivascular dense tumor-like nodularities and micronodular opacities [13]. Histopathology of tumoral lesions remains the gold standard for the diagnosis of KS, combined with immunohistochemistry for antigens of HHV-8, serology, and viral load for HHV-8 [5]. The detection of visceral lesions, GI, and lymph nodes involvements is established through a combination of diagnostic modalities including colonoscopy, esophagogastroduodenoscopy, total-body CT scan, and thorough ear nose and throat evaluation [5].

The staging of AIDS-related KS (AIDS Clinical Trials Group (ACTG)) divides patients into good or poor prognostic groups based on three parameters: the extent of the tumor, immune status, and severity of systemic illness. Disease limited to skin with minimal oral mucosa involvement produces a favorable prognosis, whereas more extensive spread tends toward a poorer prognosis. The immune status constitutes the most important prognostic factor and is reflected by the CD4 count (I0: CD4 above 200 cells/µL; I1: CD4 less than 200 cells/µL). The third prognostic factor consists of a history of opportunistic infection, thrush, constitutional B symptoms, and a Karnofsky performance scale of 70, allotting an S1 classification when present, and S0 when absent [14].

The first aim in the treatment of KS is to stop the progression of the disease [4]. Treatment approaches account for the clinical variant of KS, the extent of tumor growth, immune status, comorbidities, and symptomatology [8]. The mainstay of treatment of AIDS-related KS is the institution of HAART, which has demonstrated remarkable regression of KS solely with initiation, as well as prolonging time to treatment failure, and improved survival rates in pulmonary KS [9,15]. Despite the documented decrease in KS incidence, about 15% of HIV-infected patients, with high CD4 count and low viral load, still reportedly develop KS [16].

Localized symptomatic treatment is reserved for limited or bulky disease; however, extensive disease warrants systematic therapy [2,8], since local skin-focused therapy is unable to prevent the development of new lesions [17]. Several approaches to the treatment of localized skin disease exist and they include skin-directed therapy (cryotherapy, laser, and radiation), topical therapy (alitretinoin), and intralesional chemotherapy (vinblastine) [8]. In cases with more extensive disease, advanced or rapidly progressive KS, systematic chemotherapy, alongside HAART, is indicated, with the preferred regimen being liposomal doxorubicin [17]. Liposomal doxorubicin, a pegylated liposomal component, tends to accumulate in Kaposi lesions [7]. Other systemic treatment regimens include paclitaxel, bleomycin, vinblastine, vincristine, and etoposide [16]. Initiating HAART and chemotherapy in the early stages of diagnosis can significantly improve the five-year survival rate by up to 90% [1]. Upon the institution of HAART, some patients may experience clinical worsening of KS in the setting of KS immune reconstitution inflammatory syndrome (KS-IRIS) [8]. KS-IRIS is most frequently paradoxical, and only rarely unmasking [3]. Maintaining treatment with HAART alongside systemic adjunctive chemotherapy is indicated for the treatment of KS-IRIS [3,8].

The patients in both cases in this report developed skin lesions suggestive of KS and were both recently diagnosed to be HIV-positive prior to presentation. Initially, the first patient developed typical cutaneous lesions on the extremities along with oral mucosal involvement and already had a diagnosis of an opportunistic infection (Piringer-Kuchinka lymphadenitis). The second patient had mucocutaneous and lymph nodal involvement of KS prior to presentation. Both of their initial presentations and imaging findings were similar, and both presentations raised suspicion of the visceral involvement of KS. Detection of HHV-8 in pleural fluid established the diagnosis. The neoplastic cutaneous lesions in the first case were complicated by superimposed infection by MDR bacteria, as the patient rapidly deteriorated in the ICU, and passed away before the initiation of HAART. The ACTG staging places the first patient with a T1/I0/S1 stage, whereas the second had only one poor prognostic factor at a stage of T1/I0/S0, reflected by a more stable course in the hospital. HAART was started prior to liposomal doxorubicin therapy and displayed continued excellent clinical response after three months of follow-up.

This case report has several limitations. The findings from two single cases may not be generalizable. Additionally, there was a technical delay in procuring timely treatment for the patient in Case 1, which may have impacted the patient's outcome. A larger study is necessary to assess the effects of treatment delays on prognosis and validate these findings.

## Conclusions

We report two cases of AIDS-related visceral KS affecting the pleura and manifesting with bilateral pleural effusions in two patients with CD4 counts above 200 cells/µL. Both diagnoses were made by combining the typical skin lesions with positive HHV-8 PCR on pleural fluid. The first patient deteriorated prior to the initiation of HAART, whereas the second patient displayed notable improvement upon treatment with liposomal doxorubicin and HAART. The occurrence of pleural AIDS-related KS should be included as part of the initial differential diagnosis in HIV-positive patients presenting with respiratory symptoms, even in the absence of cutaneous lesions. HAART represents the cornerstone of treatment and prompt initiation is crucial.
